# Prognostic Significance of Carbonic Anhydrase IX Expression in Cancer Patients: A Meta-Analysis

**DOI:** 10.3389/fonc.2016.00069

**Published:** 2016-03-29

**Authors:** Simon J. A. van Kuijk, Ala Yaromina, Ruud Houben, Raymon Niemans, Philippe Lambin, Ludwig J. Dubois

**Affiliations:** ^1^Department of Radiation Oncology (MAASTRO Lab), GROW – School for Oncology and Developmental Biology, Maastricht University Medical Centre, Maastricht, Netherlands; ^2^Department of Radiation Oncology, MAASTRO Clinic, Maastricht, Netherlands

**Keywords:** cancer, carbonic anhydrase IX, hypoxia, meta-analysis, prognosis

## Abstract

Hypoxia is a characteristic of many solid tumors and an adverse prognostic factor for treatment outcome. Hypoxia increases the expression of carbonic anhydrase IX (CAIX), an enzyme that is predominantly found on tumor cells and is involved in maintaining the cellular pH balance. Many clinical studies investigated the prognostic value of CAIX expression, but most have been inconclusive, partly due to small numbers of patients included. The present meta-analysis was therefore performed utilizing the results of all clinical studies to determine the prognostic value of CAIX expression in solid tumors. Renal cell carcinoma was excluded from this meta-analysis due to an alternative mechanism of upregulation. 958 papers were identified from a literature search performed in PubMed and Embase. These papers were independently evaluated by two reviewers and 147 studies were included in the analysis. The meta-analysis revealed strong significant associations between CAIX expression and all endpoints: overall survival [hazard ratio (HR) = 1.76, 95% confidence interval (95%CI) 1.58–1.98], disease-free survival (HR = 1.87, 95%CI 1.62–2.16), locoregional control (HR = 1.54, 95%CI 1.22–1.93), disease-specific survival (HR = 1.78, 95%CI 1.41–2.25), metastasis-free survival (HR = 1.82, 95%CI 1.33–2.50), and progression-free survival (HR = 1.58, 95%CI 1.27–1.96). Subgroup analyses revealed similar associations in the majority of tumor sites and types. In conclusion, these results show that patients having tumors with high CAIX expression have higher risk of locoregional failure, disease progression, and higher risk to develop metastases, independent of tumor type or site. The results of this meta-analysis further support the development of a clinical test to determine patient prognosis based on CAIX expression and may have important implications for the development of new treatment strategies.

## Introduction

Hypoxia is a characteristic of many different types of solid tumors and is caused by an inadequate vascular supply. Hypoxic areas are characterized by low oxygen concentrations, limited nutrient supply, and an acidic extracellular environment. Hypoxia is an independent prognostic factor of poor outcome in patients (1) and decreases the efficacy of standard treatment modalities, such as surgery, chemotherapy, and radiotherapy (2–4). Many strategies are therefore being investigated to measure tumor hypoxia to predict treatment outcome and to overcome or target tumor hypoxia with newly designed treatments (5–8).

Tumor cells have adopted several mechanisms to survive the hostile conditions during hypoxia, of which one is the hypoxia-inducible factor (HIF) pathway (9, 10). Upon hypoxic conditions, the expression of the dimeric zinc-containing glycoprotein carbonic anhydrase IX (CAIX) is enhanced as a consequence of HIF stabilization (11, 12). CAIX is important in maintaining the cellular pH regulation and is located on the cell membrane where it hydrolyzes carbon dioxide, produced as a waste product during glycolysis, to bicarbonate and a proton. The bicarbonate is transported intracellularly by different proteins (e.g., anion exchangers), thereby slightly increasing the intracellular pH to promote tumor cell proliferation. The protons in turn add to an acidic extracellular environment causing extracellular matrix degradation favoring invasion, migration, and subsequent metastasis formation (12). Hypoxia-induced CAIX expression, tumor-specific expression of CAIX, and its important role in maintaining the pH balance make CAIX a promising endogenous marker of tumor hypoxia and an attractive target for anti-cancer therapies with newly designed inhibitors (6, 11, 12).

Many clinical studies investigated the prognostic value of CAIX, and a recent meta-analysis of renal cell carcinoma (RCC) concluded that high CAIX expression was associated with a better overall survival (OS) (13). By contrast, a meta-analysis in head and neck cancer patients showed high CAIX expression was associated with a decrease in both OS and disease-free survival (DFS) (14). This discrepancy can be explained by the fact that RCCs are often characterized by an inactive mutant version of the Von Hippel–Lindau (VHL) protein preventing proteasomal degradation of CAIX upon normoxia and making its expression therefore independent of hypoxia (15, 16). To the best of our knowledge, a comprehensive meta-analysis of the association between CAIX expression and treatment outcome in other tumor types has not been performed. The aim of this meta-analysis of published clinical studies is therefore to elucidate the prognostic value of CAIX expression in all solid tumor types besides RCC. In addition, current analysis has included sensitivity and subgroup analysis to be able to determine if the prognostic value of CAIX expression varies in patients with different tumor types.

## Methods

### Literature Search

**Figure 1:**
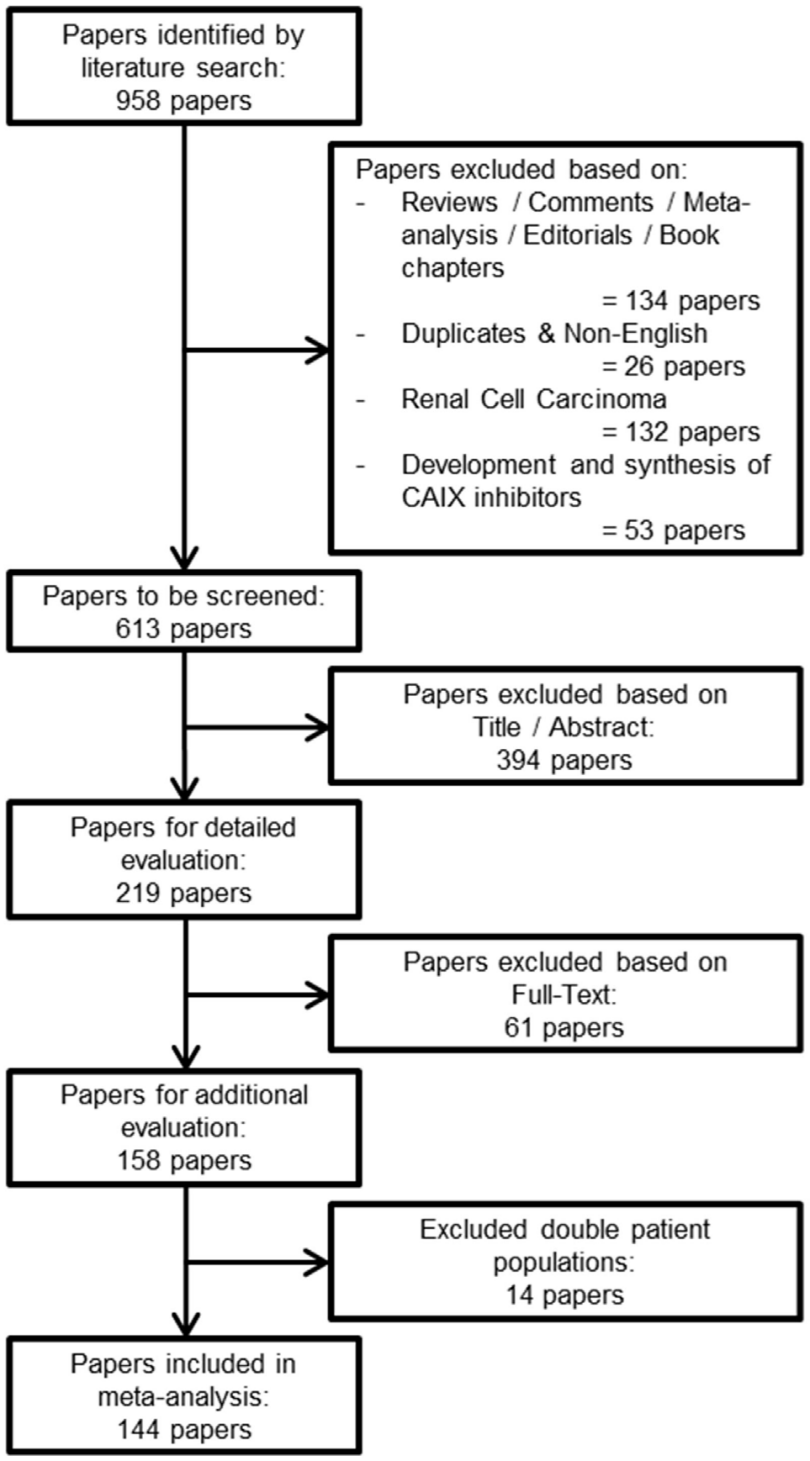
**Flowchart of selecting articles describing the association between tumoral CAIX expression and prognosis**.

The research question of this meta-analysis was defined as follows: “what is the prognostic value of tumoral CAIX expression in patients with solid tumors?” From this research question, three distinctive keywords were identified, i.e., prognosis, CAIX, and tumor. Different formulations and truncations of the keywords were tested as free text searches to see if appropriate papers could be identified. The search algorithm was applied as a free text search and consisted of the combined mention of all three keywords, in any of the formulations or truncations (Data sheet 1 in Supplementary Material). The search for literature was performed on the 31st of August 2015 in both the PubMed and Embase databases. A total of 958 papers were identified from both databases (Figure 1).

### Exclusion Criteria

From the total number of papers, 134 reviews, conference abstracts, commentaries, meta-analyses, editorials, or book chapters were excluded, as were 26 duplicates or non-English papers. From the remaining articles, 132 papers about RCC were excluded, since upregulation of CAIX in RCC is biologically different from other solid tumor types (15, 16). Furthermore, from our experience, we know that papers that describe solely the development and synthesis of CAIX inhibitors do not include patient data, and 53 papers were therefore also excluded. The total number of papers for further screening was thereby reduced to 613 (Figure 1).

### Screening of Papers

Two researchers (SK and AY) screened the remaining papers independently. The first round of screening was based on the title and abstract, whereas the second round consisted of a detailed evaluation of the full-text. Papers were evaluated based on the predetermined inclusion criteria. First, only solid primary tumors of various types were included, thereby automatically excluding hematological cancer. Second, only immunohistochemical detection of CAIX was included, because mRNA upregulation of CA9 does not fully correlate with an increase in functional protein expression, possibly due to posttranscriptional processing and/or differences in stability (17–19). Third, all endpoints were included (see below) with a minimal median follow-up of 1 year. Fourth, all treatment modalities along with experimental treatments were included. Finally, we included every human patient population without making distinction based on tumor grades or stages. Discrepancies between the included papers by both reviewers were discussed and consensus was reached on all. An additional 14 papers were excluded because their patient populations were similar or overlapping with other papers. Among these repetitive studies, the paper that was included contained the most detailed information about the patient population. A total of 144 papers were included in the meta-analysis (Figure 1).

### Data Extraction

Several different parameters, if reported, were extracted from each paper, i.e., the number and origin of patients, number of events, treatment modalities, tumor site, tumor stage, tumor type, group dichotomization, antibody supplier, expression pattern, cellular localization, and endpoints. The univariate hazard ratio (HR) was extracted to assess prognostic value of CAIX expression. When the univariate HR with corresponding 95% confidence interval (95%CI) was not reported, the method from Tierney et al. was used to estimate the HR (20). Multivariate HR was only included in the meta-analysis when the univariate HR was not reported or could not be estimated. When insufficient data were reported for estimating HR, the authors were contacted to obtain additional data.

### Quality Assessment

The methodological quality of the included papers was evaluated with an adjusted version of the Newcastle–Ottawa scale (NOS) to better suit the study design of the included papers (Data sheet 2 in Supplementary Material). The method of scoring based on awarding stars in different categories remained, however, identical. The NOS was commended in the 2011 version of the Cochrane Collaboration handbook and is an easy method to evaluate the methodological quality of cohort studies (available at http://www.ohri.ca/programs/clinical_epidemiology/oxford.asp) (21).

### Statistical Analysis and Sensitivity Analysis

Distribution and frequencies of the extracted data parameters were analyzed using SPSS (version 22). Meta-analysis was performed using R statistical software with the Metafor Library (version 1.9-8) (22). Fixed-effect modeling was performed when no statistical significant heterogeneity between studies was observed. When the heterogeneity between studies was statistically significant, random-effects modeling was applied based on the DerSimonian and Laird method (23). The assigned weight of each study in the analysis was based on its inverse variance. The following endpoints have been addressed: OS, DFS, locoregional control (LC), disease-specific survival (DSS), metastasis-free survival (MFS), and progression-free survival (PFS). Sensitivity analysis was performed by analyzing subgroups of studies separately, e.g., per tumor organ site. Funnel plots were created to visualize possible publication bias or heterogeneity between studies. Asymmetric funnel plots and studies outside the funnel plot suggest heterogeneity between them and/or publication bias (21). *p*-Values <0.05 were considered as statistically significant.

## Results

This meta-analysis encompassed a total number of 24,523 patients across 147 independent studies. Many studies included only a small number of patients (median per study 93, range 15–3630) with a median follow-up time between 12.6 months and 13.9 years and are often inconclusive, which underlines the need for a meta-analysis. All papers were published between 2001 and 2015 of which approximately 50% were published after 2010. The majority of the included studies treated patients with surgery alone (36.7%) or in combination with either chemotherapy (8.8%) or standard radiotherapy (8.8%), or the combination of all three modalities (23.1%). Single radiotherapy treatment or combined with chemotherapy was reported in 5.4 and 6.1% of the papers, respectively. In 4.8% of the studies, a form of experimental treatment was administered, including experimental radiotherapy (24–28), hormonal treatment (29), and VEGF-targeted therapy (30). Most of the studies reported on head and neck cancer patients (21.8%) followed by breast (16.3%) and brain cancer patients (10.2%). By contrast, cancers of the adrenal gland, the cartilage, and the penis were only described once.

Immunohistochemical staining of CAIX was predominantly performed using the M75 antibody (46.3%) targeting the proteoglycan domain of CAIX (31, 32). Other studies used anti-CAIX antibodies obtained from different suppliers. A membranous expression of CAIX was described in 46.3% of the studies, although cytoplasmatic staining or a combination of the two was also reported (4.8 and 17.7%, respectively). Nuclear staining was only reported in one paper, whereas the rest did not state the staining localization. Different quantification methods and thresholds have been applied to stratify patients into groups with low and high tumoral CAIX expression. Taken together, 33.9% of the total tumors were classified as expressing high levels of CAIX.

**Figure 2:**
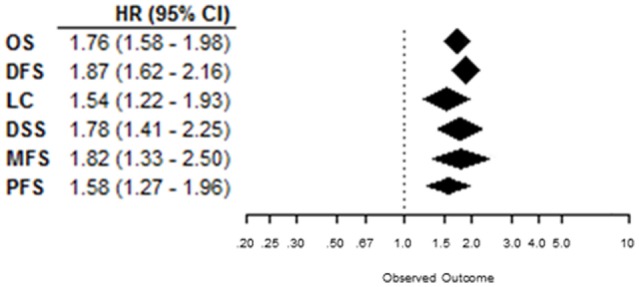
**Summary plot of the overall HRs from each endpoint analyzed**. Symbols represent the HR with 95%CI, and dashed line indicates no association between CAIX expression and prognosis.

Overall, patients suffering from tumors with high CAIX expression had a worse treatment outcome (Figure 2). This association was strong and significant for all endpoints. The negative association of CAIX expression with outcome was dominant for DFS and weaker for LC. Systematic heterogeneity in the present meta-analysis as demonstrated by an asymmetric funnel plot (21) for most of the endpoints (Image 1 in Supplementary Material) can at least in part be attributed to the considerable variation in tumor types and sites across the studies. Therefore, in addition, subgroup analysis based on organ site of the tumor was performed. The results of the subgroup analysis demonstrate a significant prognostic value of CAIX in most of the cancer types investigated (see below).

### Overall Survival

**Figure 3:**
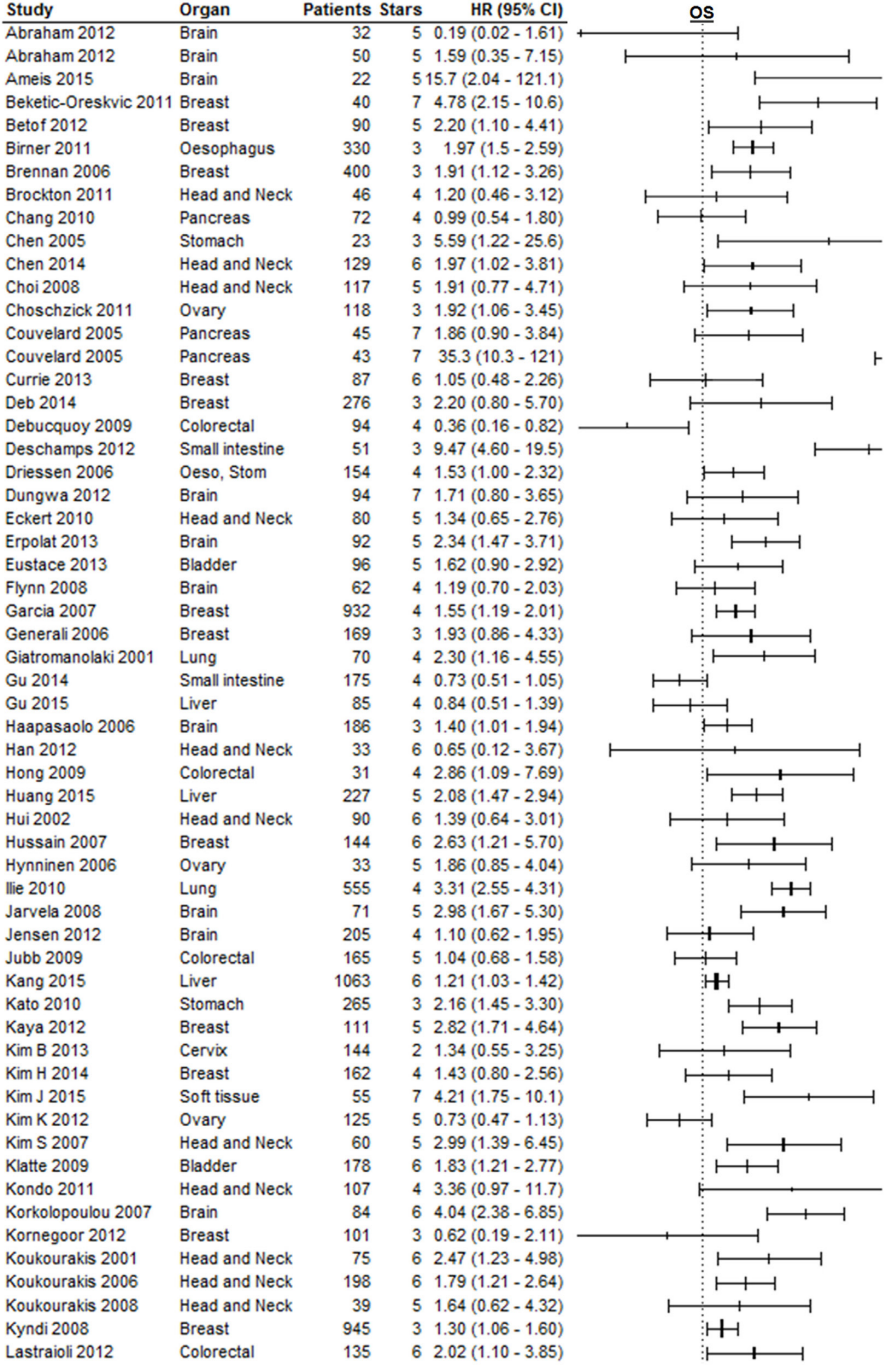

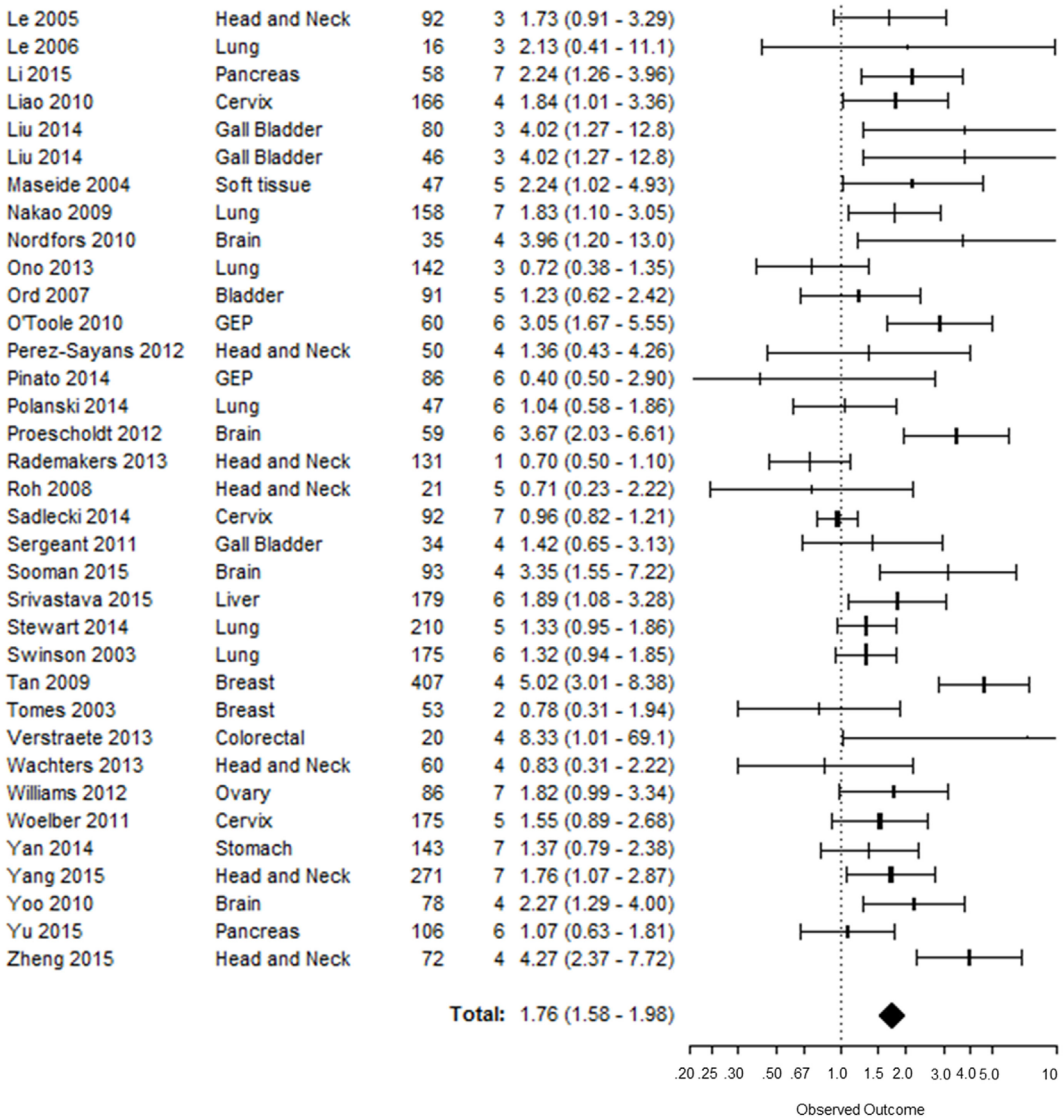
**Forest plot of the papers describing the association between CAIX expression and OS**. Horizontal bars represent HR with corresponding 95%CI. Symbol size represents the assigned weight of the study. The overall HR with 95%CI is visualized with the diamond shape. Dashed line indicates no association between CAIX and prognosis. Esop, Stom, esophagus and stomach; GEP, gastroenteropancreatic tract (25–27, 29, 30, 44–129).

Effect of pretreatment expression of CAIX on OS could be evaluated in 104 studies. The complete data to estimate the HR could not be retrieved from 11 papers and were therefore not included in the analysis [Table 1 in Supplementary Material (33–43)]. Overall, high CAIX expression was associated with a worse OS (HR = 1.76, 95%CI 1.58–1.98, *p* < 0.0001, Figure 3). Subgroup analysis of the different organ sites revealed a similar significant association between tumoral CAIX expression and OS in 11 organ sites: bladder (HR = 1.64, 95%CI = 1.21–2.22), brain (HR = 2.18, 95%CI 1.60–2.96), breast (HR = 1.90, 95%CI = 1.45–2.50), esophagus (HR = 1.97, 95%CI 1.50–2.60), gall bladder (HR = 2.35, 95%CI 1.33–4.15), gastroenteropancreatic tract (HR = 2.57, 95%CI 1.45–4.56), head and neck (HR = 1.66, 95%CI 1.29–2.13), lung (HR = 1.57, 95%CI 1.06–2.33), pancreas (HR = 2.37, 95%CI 1.04–5.43), soft tissue (HR = 2.97, 95%CI 1.65–5.34), and the stomach (HR = 1.92, 95%CI 1.39–2.67). The other six organ sites show a similar trend with worse OS, albeit not statistically significant (Table 1). Similar results were often, but not always, observed for different tumor types per organ site (Table 2 in Supplementary Material). A hypoxia-associated perinecrotic staining pattern was reported in 16 of these studies, whereas a diffuse staining pattern was reported in 3 papers. Interestingly, both patterns of CAIX expression significantly associated with OS (perinecrotic: HR = 1.99, 95%CI 1.60–2.48; diffuse: HR = 1.77, 95%CI 1.22–2.56). These results suggest that the expression pattern of CAIX does not affect its prognostic value.

**Table 1:** **Results of subgroup meta-analyses of different organ sites reported**.

Organ site	OS	DFS	LC	DSS	MFS	PFS
Bladder	**1.64 (1.21–2.22)**	**2.63 (1.56–4.40)**	0.88 (0.40–1.90)	0.82 (0.47–1.4)		0.68 (0.21–2.20)
Brain	**2.18 (1.60–2.96)**					1.44 (0.91–2.27)
Breast	**1.90 (1.45–2.50)**	**1.74 (1.34–2.27)**	1.37 (0.95–1.96)	**1.75 (1.28–2.38)**	**1.76 (1.13–2.74)**	**1.88 (1.13–3.10)**
Cartillage					**6.46 (2.05–20.0)**	
Cervix	1.11 (0.91–1.35)	1.12 (0.75–1.68)	1.17 (0.74–1.87)	**2.19 (1.29–3.70)**	**2.37 (1.35–4.10)**	1.76 (0.99–3.10)
Colorectal	1.41 (0.67–2.98)	**3.31 (1.23–8.89)**	3.33 (1.76–6.30)	1.31 (0.18–9.41)	**5.17 (2.07–13.0)**	**2.38 (1.06–5.56)**
Esophagus	**1.97 (1.50–2.60)**	**2.70 (2.08–3.50)**		**2.78 (1.56–5.00)**		
Esop, Stom	1.53 (1.00–2.30)					
Gall Bladder	**2.35 (1.33–4.15)**					
GEP	**2.57 (1.45–4.56)**					
H&N	**1.66 (1.29–2.13)**	**1.98 (1.51–2.61)**	**1.54 (1.12–2.12)**	**2.21 (1.12–4.36)**	0.77 (0.27–2.26)	**1.62 (1.01–2.59)**
Liver	1.41 (0.98–2.03)	**1.51 (1.26–1.81)**	**1.39 (1.09–4.10)**			
Lung	**1.57 (1.06–2.33)**	**1.87 (1.27–2.74)**		1.75 (0.59–5.15)		
Ovary	1.42 (0.82–2.45)					1.24 (0.67–2.30)
Pancreas	**2.37 (1.04–5.43)**	2.98 (0.56–15.9)		**1.49 (1.07–2.10)**		
Penis		1.35 (0.55–3.30)				
Small Intestine	2.58 (0.21–31.8)					
Soft tissue	**2.97 (1.65–5.34)**	**3.41 (1.58–7.30)**		**1.65 (1.11–2.45)**	1.65 (0.72–3.80)	
Stomach	**1.92 (1.39–2.67)**	1.27 (0.77–2.10)				
Vulva		1.52 (0.79–2.90)		1.34 (0.67–2.70)	**2.25 (1.42–3.60)**	

*The overall HR (with 95%CI) is shown. When HR was available from only one paper, the values were adopted from that single paper. Bold numbers indicate statistical significant associations between CAIX expression and prognosis (*p* < 0.01)*.

*Esop, Stom, esophagus and stomach; GEP, gastroenteropancreatic tract*.

### Disease-Free Survival

**Figure 4:**
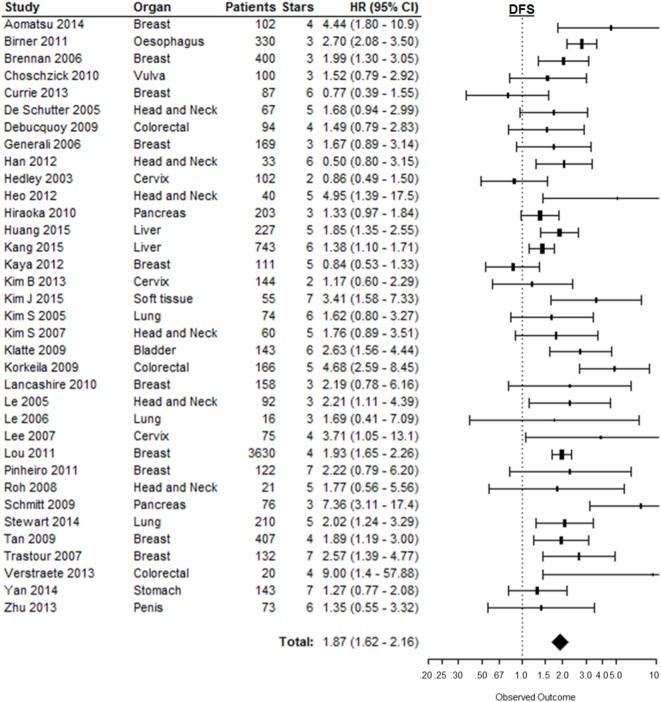
**Forest plot of the papers describing the association between CAIX expression and DFS**. Horizontal bars represent HR with corresponding 95%CI. Symbol size represents the assigned weight of the study. The overall HR with 95%CI is visualized with the diamond shape. Dashed line indicates no association between CAIX and prognosis (29, 33, 34, 48, 57, 59, 68, 73, 74, 82, 84, 85, 87, 89, 90, 97, 98, 112, 117, 119, 121, 125, 131–143).

A total of 40 from the selected 147 studies investigated the association between CAIX expression and DFS. Five studies could not be included in this analysis due to incomplete reporting [Table 1 in Supplementary Material (39, 40, 43, 124, 130)]. Based on 35 studies, high CAIX expression was statistically significantly associated with a decreased DFS (HR = 1.87, 95%CI 1.62–2.16, *p* < 0.001) (Figure 4). Subgroup analysis based on organ site of the tumor showed that high CAIX expression was significantly associated with shorter DFS in bladder (HR = 2.63, 95%CI 1.56–4.40), breast (HR = 1.74, 95%CI 1.34–2.27), colorectal (HR = 3.31, 95%CI 1.23–8.89), esophagus (HR = 2.70, 95%CI 2.08–3.50), head and neck (HR = 1.98, 95%CI 1.51–2.61), liver (HR = 1.51, 95%CI 1.26–1.81), lung (HR = 1.87, 95%CI 1.27–2.74), and soft tissue tumors (HR = 3.41, 95%CI 1.58–7.30). By contrast, no significant association with DFS was observed for tumors in the cervix (HR = 1.12, 95%CI 0.75–1.68), pancreas (HR = 2.98, 95%CI 0.56–15.9), penis (HR = 1.35, 95%CI 0.55–3.30), stomach (HR = 1.27, 95%CI 0.77–2.10), and vulva (HR = 1.52, 95%CI 0.79–2.90) (Table 1). A similar trend was observed for all different tumor types per organ sites (Table 2 in Supplementary Material).

### Locoregional Control

**Figure 5:**
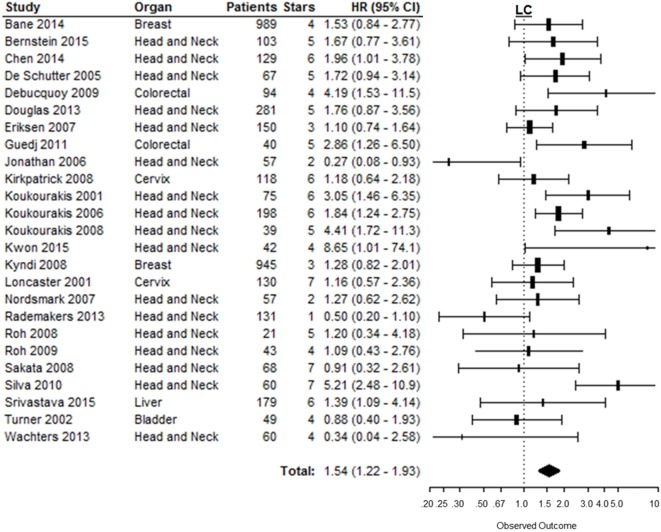
**Forest plot of the papers describing the association between CAIX expression and LC**. Horizontal bars represent HR with corresponding 95%CI. Symbol size represents the assigned weight of the study. The overall HR with 95%CI is visualized with the diamond shape. Dashed line indicates no association between CAIX and prognosis (24–28, 52, 59, 94, 95, 112, 116, 122, 133, 144–155).

The risk of locoregional relapse associated with CAIX expression was evaluated in 25 studies in 6 different organ sites. Figure 5 shows the overall LC outcome, which indicates that patients with high tumoral CAIX expression have a higher risk of locoregional recurrences than patients with low expression of CAIX in tumors (HR = 1.54, 95%CI 1.22–1.93, *p* = 0.0002). The negative association between high CAIX expression in tumors and worse LC remained significant in head and neck (HR = 1.54, 95%CI 1.12–2.12) and liver tumors (HR = 1.39, 95%CI 1.09–4.10) (Table 1). A similar association was observed in most of the tumor types per organ sites (Table 2 in Supplementary Material).

### Disease-Specific Survival

**Figure 6:**
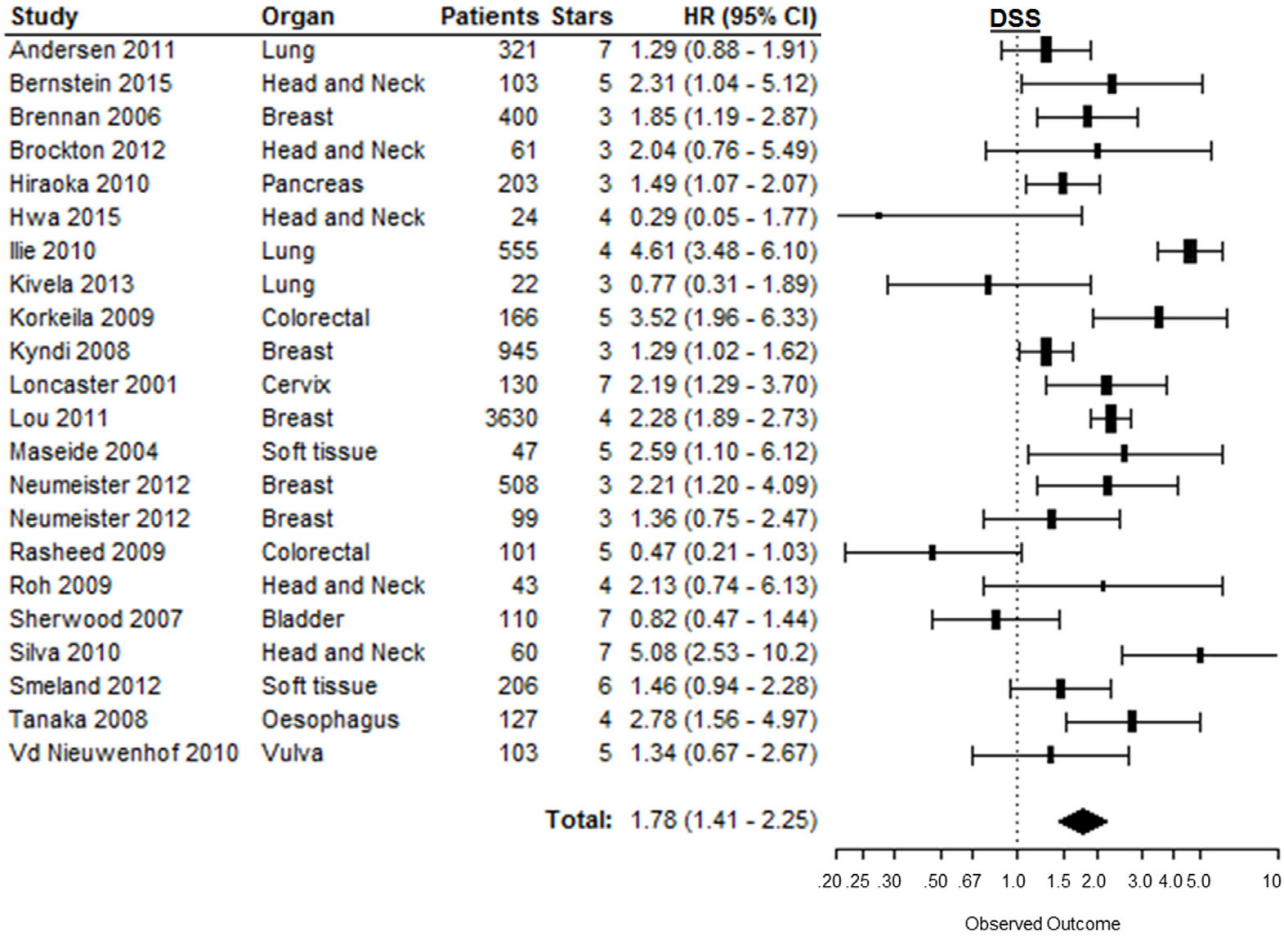
**Forest plot of the papers describing the association between CAIX expression and DSS**. Horizontal bars represent HR with corresponding 95%CI. Symbol size represents the assigned weight of the study. The overall HR with 95%CI is visualized with the diamond shape. Dashed line indicates no association between CAIX and prognosis (29, 78, 95, 102, 136, 138, 140, 144, 150, 152, 154, 156–165).

Disease-specific survival was reported in 23 studies, of which 1 study provided incomplete data to estimate the HR [Table 1 in Supplementary Material (43)]. In the remaining 22 studies, patients suffering from tumors with high CAIX expression had a significantly shorter DSS (HR = 1.78, 95%CI 1.41–2.25, *p* < 0.0001) (Figure 6). Subgroup analyses by organ site revealed significant associations between high CAIX expression and worse DSS in tumors of the breast (HR = 1.75, 95%CI 1.28–2.38), cervix (HR = 2.19, 95%CI 1.29–3.70), esophagus (HR = 2.78, 95%CI 1.56–5.00), head and neck (HR = 2.21, 95%CI 1.12–4.36), pancreas (HR = 1.49, 95%CI 1.07–2.10), and soft tissue (HR = 1.65, 95%CI 1.11–2.45) (Table 1). Subgroup analyses of the tumor types per organ sites revealed a worse DSS to be associated with high CAIX expression in the majority of tumor types (Table 2 in Supplementary Material).

### Metastasis-Free Survival

**Figure 7:**
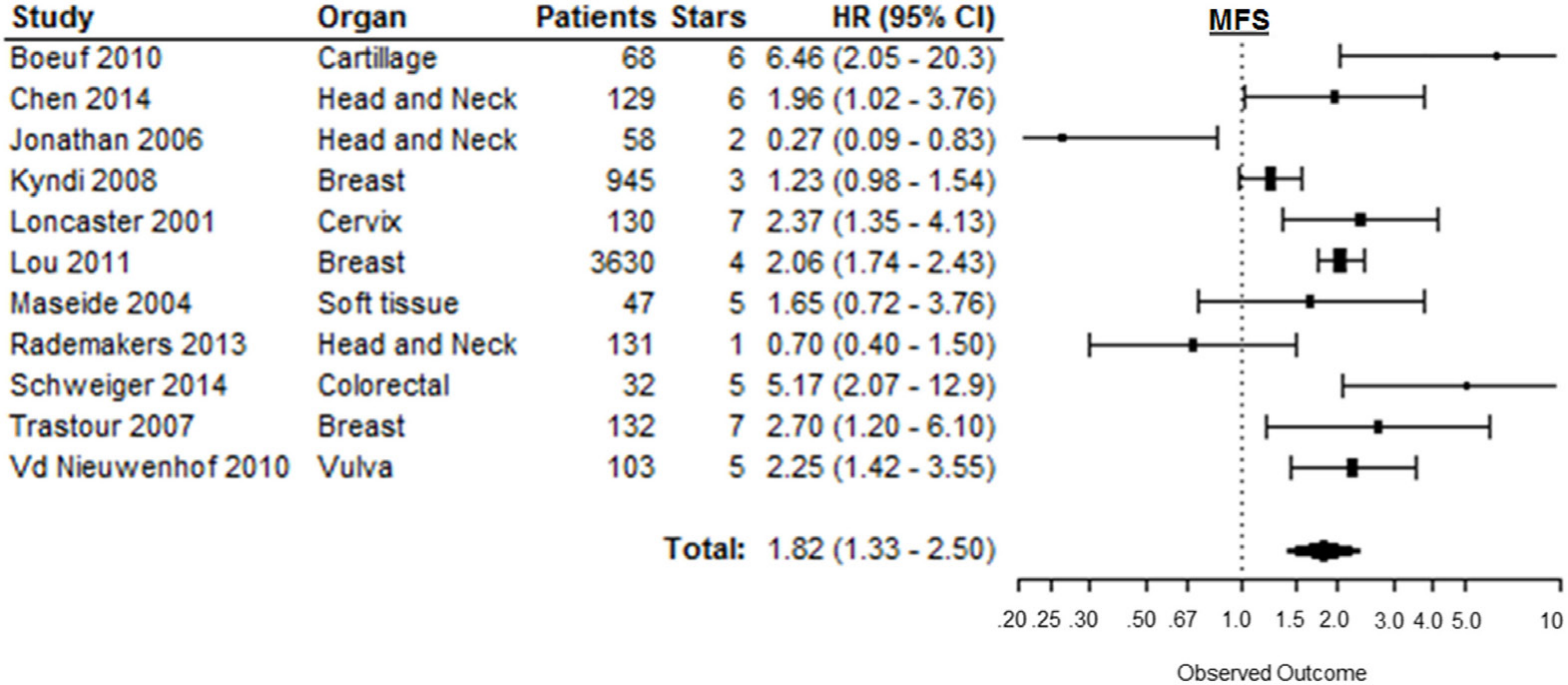
**Forest plot of the papers describing the association between CAIX expression and MFS**. Horizontal bars represent HR with corresponding 95%CI. Symbol size represents the assigned weight of the study. The overall HR with 95%CI is visualized with the diamond shape. Dashed line indicates no association between CAIX and prognosis (24, 27, 34, 52, 95, 102, 140, 34, 150, 165, 167, 168).

Metastasis-free survival was reported in 12 of the 147 included studies. Based on 11 of these studies, high CAIX expression was significantly associated with a shorter MFS (HR = 1.82, 95%CI 1.33–2.50, *p* = 0.0002) [Figure 7; Table 1 in Supplementary Material (166)]. Subgroup analyses of the different organ sites of the tumors, independent of tumor types, revealed high CAIX expression to be significantly associated with a worse MFS in most of the organ sites reported, i.e., breast (HR = 1.76, 95%CI 1.13–2.74), cartilage (HR = 6.46, 95%CI 2.05–20.0), cervix (HR = 2.37, 95%CI 1.35–4.10), colorectal (HR = 5.17, 95%CI 2.07–13.0), and vulva (HR = 2.25, 95%CI 1.42–3.60), but not in head and neck (HR = 0.77, 95%CI 0.27–2.26) and soft tissue cancers (HR = 1.65, 95%CI 0.72–3.80) (Table 1). Interestingly, one study reported a significant positive association between high CAIX expression and better MFS in squamous cell carcinoma of the head and neck (HR = 0.27, 95%CI 0.09–0.80) (Table 2 in Supplementary Material), which may be attributed to the hypoxia-modifying component of the treatment (24).

### Progression-Free Survival

**Figure 8:**
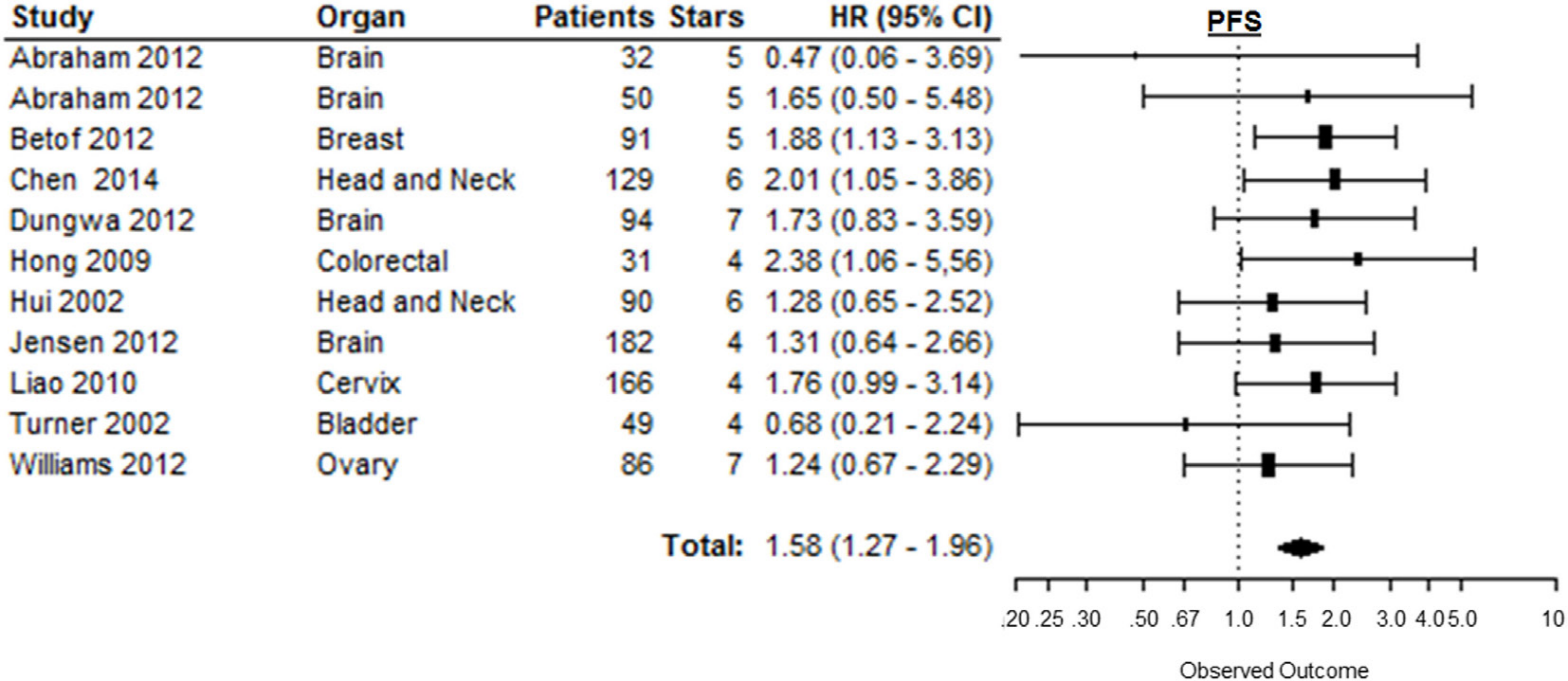
**Forest plot of the papers describing the association between CAIX expression and PFS**. Horizontal bars represent the HR with corresponding 95%CI. Symbol size represents the assigned weight of the study. The overall HR with 95%CI is visualized with the diamond shape. Dashed line indicates no association between CAIX and prognosis (30, 44, 47, 52, 62, 75, 80, 100, 123, 155).

Eleven out of 12 studies could be included to estimate the risk of disease progression after treatment based on CAIX expression in tumors [Table 1 in Supplementary Material (169)]. Similar to the other endpoints, PFS was significantly shorter in patients with tumors expressing high levels of CAIX (HR = 1.58, 95%CI 1.27–1.96, *p* < 0.0001) (Figure 8). Subgroup analyses per organ site revealed that the association with PFS only remained statistically significant in breast (HR = 1.88, 95%CI 1.13–3.10), colorectal (HR = 2.38, 95%CI 1.06–5.56), and head and neck tumors (HR = 1.62, 95%CI 1.01–2.59) (Table 1). The subgroup analyses of tumor types per organ site showed similar associations between high CAIX expression and a worse PFS (Table 2 in Supplementary Material).

### High-Quality Papers

This meta-analysis used an adjusted version of the NOS to evaluate the quality of a study. The scores of this quality assessment ranged between 1 and 7 stars, i.e., the maximum, awarded per study. Approximately half of the studies (52.4%) were considered as high-quality studies, i.e., with a number of stars greater or equal to the median (5 stars). Meta-analysis of only the high-quality studies revealed significant prognostic values of CAIX expression for OS (HR = 1.81, 95%CI 1.57–2.09, *n* = 50), DFS (HR = 1.81, 95%CI 1.47–2.23, *n* = 18), DSS (HR = 1.71, 95%CI 1.16–2.51, *n* = 10), and PFS (HR = 1.59, 95%CI 1.21–2.07, *n* = 7). For both LC (HR = 1.90, 95%CI 1.58–2.30, *n* = 14) and MFS (HR = 2.47, 95%CI 1.92–3.19, *n* = 7), the association with CAIX expression became even stronger when only high-quality studies were included.

## Discussion

Many clinical studies investigated the prognostic association of CAIX expression with treatment outcome. Most of these studies, however, include only limited numbers of patients and remain inconclusive. This current meta-analysis is the first complete overview of all reported clinical studies investigating the impact of pretreatment CAIX expression in solid tumors on prognosis. Overall, these results clearly show that high CAIX expression is an adverse prognostic marker in solid tumors, irrespectively of the endpoint evaluated, as summarized in Figure 2. A strong association between high CAIX expression and poor prognosis was also found in the majority of different tumor sites, supporting an important role of CAIX in disease progression and treatment resistance in many cancer types.

The papers included in the current meta-analysis were all published between 2001 and 2015, which is likely attributed to the identification of the hypoxic responsive element in the promotor region of *ca9* in the end of 2000 (170). This study identified a direct link between CAIX expression and its hypoxic upregulation through HIF stabilization. This crucial finding encouraged research to evaluate CAIX as an endogenous marker of tumor hypoxia, a known biological factor of therapy resistance (2–4). Nevertheless, because alternative mechanisms can also regulate CAIX expression, e.g., *via* PI3K (171) or the unfolded protein response (10, 172), tumoral CAIX expression may not accurately identify hypoxic tumors. Apart from the hypoxia-associated mechanisms underlying resistance of tumor cells to several treatment modalities, CAIX can directly affect cancer prognosis as its main function is to maintain the balance between intracellular and extracellular pH, thereby generating an acidic extracellular microenvironment (11, 12). This is supported by data demonstrating that CAIX is involved in promoting tumorigenesis and leads to a more aggressive phenotype of tumor cells (173). This can partly be explained by the association between CAIX expression and the induction of tumor cell migration and invasion, which could be caused by the reduction in extracellular pH (174–176). In addition, cancer stem cell markers also appear to be enriched in the CAIX expressing population of tumor cells (57, 177). The important role of CAIX, either directly or indirectly, in cancer prognosis is also supported by the results of the current meta-analysis, which shows that tumors with high CAIX expression have higher risk of locoregional failure, disease progression, and higher risk to develop metastasis. Other proton exchangers and transporters have been shown preclinically and clinically to play an important role in the regulation of cellular pH homeostasis promoting survival and invasion as well as causing treatment resistance (178–180). Therefore, assessment of several major pH regulators in tumors prior and/or during therapy may represent a more powerful prognostic and predictive biomarker as well as important targets for new anti-cancer treatments, which warrants further investigations.

A meta-analysis usually overestimates its results because of selective reporting and publication bias (21). This meta-analysis identified a total of 147 studies reported in 144 papers of which 15 could not be included in final analysis because the HR could not be estimated due to incomplete reporting (33–43, 124, 130, 166, 169). Non-significant association between CAIX and outcome was found in these studies (Table 1 in Supplementary Material). Including these 15 papers in the analysis might therefore decrease the magnitude of the prognostic values of CAIX expression reported here. This overestimation can be further increased by publication bias, i.e., when negative associations are not published at all and can therefore not be identified and included in this meta-analysis. Nevertheless, since the prognostic value of CAIX expression was highly statistically significant, we believe that the possible effect of publication bias on this association is minimal.

The different staining and scoring methods used in the included papers to quantify CAIX expression might be an additional source of bias. Visual quantification was used in the majority of the reports and could either be based on staining intensity, the number of stained cells, or a combination of both. In addition, different thresholds have been used to dichotomize patients based on their CAIX expression. This discrepancy in methods is one of the reasons of significant heterogeneity between studies, which therefore requires the use of a random-effect model in the meta-analysis (181, 182). Additionally, tissue microarrays (TMAs) are used in the majority of included papers to visualize and quantify CAIX expression, even though TMAs may underestimate the actual expression levels of the protein (183). The use of TMAs might therefore bias the prognostic value of CAIX when CAIX expression levels are dichotomized erroneous. Furthermore, this meta-analysis is limited by difficulties in obtaining homogenous endpoints and by non-uniform observation times, although most of the data are based on reports with a median follow-up of more than 1 year.

To identify possible bias in a selected study, an adjusted version of the NOS was used, which is a quick and easy method to assess the quality of studies that has been commended in the Cochrane handbook (21). However, the validity and reproducibility of the NOS have been questioned because of the subjective interpretation of certain criteria, which require detailed guidelines to obtain a better inter-rater agreement (184–186). The test–retest reliability of the NOS is, however, better, which allows for a single reviewer to continuously use uniform criteria while rating papers (185). When only high-quality papers, i.e., those with minimal bias, were included in our analyses, there was no significant difference in the results as compared with all studies included.

It remains impossible to eliminate every source of bias in a meta-analysis. Nevertheless, the high statistical significance of the results presented here clearly show that CAIX expression is associated with worse prognosis in a global patient population and in the majority of tumor sites. These findings are similar to the results of the meta-analysis in head and neck cancer (14), but different from RCC (13) due to the alternative mechanism of CAIX upregulation in RCC (15, 16). New treatment options are currently being developed to specifically inhibit CAIX function (6, 187), of which one is currently in a Phase I clinical trial (NCT02215850). These types of compounds might prove to be beneficial for the specific treatment of tumors with high CAIX expression. The results of this meta-analysis further support the development of a clinical test to determine patient prognosis based on CAIX expression, although a standardized protocol remains to be developed and validated.

## Author Contributions

Study was conceived and designed by SK, AY, RN, PL, and LD. Screening of papers and data extraction was performed by SK and AY. Statistical analyses were performed by RH. Writing of the first draft of the manuscript was performed by SK. AY, RH, RN, PL, and LD contributed to the writing of the manuscript.

## Conflict of Interest Statement

The authors declare that the research was conducted in the absence of any commercial or financial relationships that could be construed as a potential conflict of interest.

## Abbreviations

95%CI, 95% confidence interval; CAIX, carbonic anhydrase IX; DFS, disease-free survival; DSS, disease-specific survival; HIF, hypoxia-inducible factor; HR, hazard ratio; LC, locoregional control; MFS, metastasis-free survival; NOS, Newcastle–Ottawa scale; OS, overall survival; PFS, progression-free survival; RCC, renal cell carcinoma; TMAs, tissue microarrays; VHL, Von Hippel–Lindau protein.
