# Supplementary material for: Prognostic Significance of Carbonic Anhydrase IX Expression in Cancer Patients: A Meta-Analysis
Source: Front Oncol. 2016 Mar 29;6:69. doi: 10.3389/fonc.2016.00069 (PMC4810028; doi:10.3389/fonc.2016.00069)
Supplement: Supplementary file 1 [file Table_1.pdf]

Supplementary Table S1. Table of papers that reported inadequate data to estimate the HR. A brief description of the available data is described in the comments.

Study	Organ	Patients	Comments
**OS papers**			
Blank 2010	Adrenal Gland	111	"The survival depending on CA-9 offered no significant trend."
Cleven 2007	Colorectal	133	"CA9 epithelial expression had no impact on patient survival."
Huber 2015	Oesophagus	112	Median survival 20 vs 20 months for CAIX high and low, respectively
Jung 2013	Stomach	193	Mean survival time 39.1 vs 39.0 months for CAIX high and low (p=0.705)
Lancashire 2010	Breast	158	Chi-squared (2.976) with p-value (0.085) reported for OS.
Lee-Kong 2012	Colorectal	85	Univariate HR reported (1.27, p=0.01), no 95% CI could be estimated.
Noh 2014	Breast	334	P-value reported (0.392), without clear description of outcome.
Preusser 2005	Brain	84	"CA9 no significant influence on patient survival" (p=0.1065).
Seeber 2010	Cervix	93	"CAIX expression was not correlated with survival".
Trastour 2007	Breast	132	DFS data available, for OS only multivariate p-value shown (p=0.2).
Winter 2006	Head and Neck	149	"CA9 expression was not associated with different OS (p=0.3)."
**DFS papers**			
Lee-Kong 2012	Colorectal	85	Univariate HR reported (1.13, p=0.10), no 95% CI could be estimated.
Noh 2014	Breast	334	P-value reported (0.985), without clear description of outcome.
Rajaganeshan 2009	Colorectal	55	"A significant trend to worse DFS was not observed."
Winter 2006	Head and Neck	149	"CA9 was not associated with a survival difference for DFS (p=0.2)."
Woelber 2011	Cervix	175	"Association CAIX and survival failed statistical significance (p=0.220)."
**DSS papers**			
Winter 2006	Head and Neck	149	"CA9 was not associated with a survival difference for DSS (p=0.1)."
**MFS papers**			
Doyen 2014	Breast	770	No association reported between CAIX expression and MFS.
**PFS papers**			
Grigsby 2007	Cervix	15	Only p-value reported for univariate survival analysis (p=0.86).
