# Supplementary material for: Prognostic Significance of Carbonic Anhydrase IX Expression in Cancer Patients: A Meta-Analysis
Source: Front Oncol. 2016 Mar 29;6:69. doi: 10.3389/fonc.2016.00069 (PMC4810028; doi:10.3389/fonc.2016.00069)
Supplement: Supplementary file 2 [file Table_2.pdf]

Supplementary Table S2. Table with the results from the subgroup meta-analysis of the tumor types per organ sites. The corresponding number of papers included in each analysis is shown. When HR was available from only one paper, the values were adopted from that single paper.. Bold numbers indicate statistical significant associations between CAIX expression and prognosis ($p<0.01$). SCC - squamous cell carcinoma. GBM - glioblastoma multiform.

Organ and Tumor Type	OS	DFS	LC	DSS	MFS	PFS
**Bladder**	**1.64 (1.21 - 2.22)**<br>n=3	**2.63 (1.56 - 4.40)**<br>n=1	0.88 (0.40 - 1.90)<br>n=1	0.82 (0.47 - 1.40)<br>n=1		0.68 (0.21 - 2.20)<br>n=1
Carcinoma	**1.83 (1.21 - 2.80)**<br>n=1	**2.63 (1.56 - 4.40)**<br>n=1				
Transitional & SCC				0.82 (0.47 - 1.40)<br>n=1		
Transitional Cell Carcinoma	1.44 (0.92 - 2.24)<br>n=2		0.88 (0.40 - 1.90)<br>n=1			0.68 (0.21 - 2.20)<br>n=1
**Brain**	**2.18 (1.60 - 2.96)**<br>n=14					1.44 (0.91 - 2.27)<br>n=4
Anaplastic Oligodendroglioma	0.19 (0.02 - 1.60)<br>n=1					0.47 (0.06 - 3.7)<br>n=1
Astrocytoma	**2.28 (1.19 - 4.37)**<br>n=3					
Astrocytoma & GBM	**2.34 (1.47 - 3.70)**<br>n=1					
Glial Tumors	**3.35 (1.55 - 7.20)**<br>n=1					
GBM	2.08 (0.69 - 6.26)<br>n=2					
Pediatric Brain Tumors	**3.96 (1.20 - 13.0)**<br>n=1					
Mengioma	1.10 (0.62 - 2.00)<br>n=1					1.31 (0.64 - 2.70)<br>n=1
Neuroblastoma	4.20 (0.50 - 35.5)<br>n=2					1.73 (0.83 - 3.60)<br>n=1
Oligodendroglioma	**2.75 (1.61 - 4.71)**<br>n=2					1.65 (0.50 - 5.50)<br>n=1
**Breast**	**1.90 (1.45 - 2.50)**<br>n=14	**1.74 (1.34 - 2.27)**<br>n=10	1.37 (0.95 - 1.96)<br>n=2	**1.75 (1.28 - 2.38)**<br>n=5	**1.76 (1.13 - 2.74)**<br>n=3	**1.88 (1.13 - 3.10)**<br>n=1
Adenocarcinoma		**2.57 (1.39 - 4.80)**<br>n=1			**2.70 (1.20 - 6.10)**<br>n=1	
Cancer	**2.02 (1.48 - 2.76)**<br>n=9	**1.67 (1.26 - 2.23)**<br>n=9	1.37 (0.95 - 1.96)<br>n=2	**1.83 (1.29 - 2.58)**<br>n=4	1.60 (0.97 - 2.65)<br>n=2	**1.88 (1.13 - 3.10)**<br>n=1
Ductal Carcinoma	1.76 (0.68 - 4.58)<br>n=3					

Organ and Tumor Type	OS	DFS	LC	DSS	MFS	PFS
Male Carcinoma	1.37 (0.64 - 2.90) n=2					
Triple Negative Breast Cancer				1.36 (0.75 - 2.50) n=1		
**Cartillage**					**6.46 (2.05 - 20.0)** n=1	
Chrondrosarcoma					**6.46 (2.05 - 20.0)** n=1	
**Cervix**	1.11 (0.91 - 1.35) n=4	1.12 (0.75 - 1.68) n=3	1.17 (0.74 - 1.87) n=2	**2.19 (1.29 - 3.70)** n=1	**2.37 (1.35 - 4.10)** n=1	1.76 (0.99 - 3.10) n=1
Cancer	**1.61 (1.11 - 2.33)** n=3	1.12 (0.75 - 1.68) n=3	1.18 (0.64 - 2.20) n=1			1.76 (0.99 - 3.10) n=1
Endometrial Cancer	0.96 (0.82 - 1.20) n=1					
SCC			1.16 (0.57 - 2.40) n=1	**2.19 (1.29 - 3.70)** n=1	**2.37 (1.35 - 4.10)** n=1	
**Colorectal**	1.41 (0.67 - 2.98) n=5	**3.31 (1.23 - 8.89)** n=3	3.33 (1.76 - 6.30) n=2	1.31 (0.18 - 9.41) n=2	**5.17 (2.07 - 13.0)** n=1	**2.38 (1.06 - 5.56)** n=1
Adenocarcinoma	1.20 (0.52 - 2.76) n=4	1.80 (0.98 - 3.31) n=2	3.33 (1.76 - 6.30) n=2	0.47 (0.21 - 1.00) n=1		
Cancer	**2.86 (1.09 - 7.70)** n=1	**4.68 (2.59 - 8.50)** n=1		**3.52 (1.96 - 6.30)** n=1	**5.17 (2.07 - 13.0)** n=1	**2.38 (1.06 - 5.56)** n=1
**Esophagus**	**1.97 (1.50 - 2.60)** n=1	**2.70 (2.08 - 3.50)** n=1		**2.78 (1.56 - 5.00)** n=1		
Cancer	**1.97 (1.50 - 2.60)** n=1	**2.70 (2.08 - 3.50)** n=1				
SCC				**2.78 (1.56 - 5.00)** n=1		
**Esophagus, Stomach**	1.53 (1.00 - 2.30) n=1					
Adenocarcinoma	1.53 (1.00 - 2.30) n=1					
**Gall Bladder**	**2.35 (1.33 - 4.15)** n=3					
Adenocarcinoma	**4.02 (1.27 - 13.0)** n=1					
Cancer	1.42 (0.65 - 3.10) n=1					
(Adeno)SCC	**4.02 (1.27 - 13.0)** n=1					
**Gastro-Entero-Pancreatic**	**2.57 (1.45 - 4.56)** n=2					
Neuroendocrine Tumor	**2.57 (1.45 - 4.56)** n=2					

Organ and Tumor Type	OS	DFS	LC	DSS	MFS	PFS
**Head and Neck**	**1.66 (1.29 - 2.13)** n=18	**1.98 (1.51 - 2.61)** n=6	**1.54 (1.12 - 2.12)** n=17	**2.21 (1.12 - 4.36)** n=5	0.77 (0.27 - 2.26) n=3	**1.62 (1.01 - 2.59)** n=2
Larynx SCC	0.72 (0.48 - 1.09) n=2		1.13 (0.38 - 3.39) n=4		0.70 (0.40 - 1.50) n=1	
Larynx & Pharynx SCC			1.20 (0.84 - 1.71) n=2	**2.31 (1.04 - 5.10)** n=1		
Oral Cavity SCC	**2.24 (1.70 - 2.95)** n=7	**2.23 (1.22 - 4.09)** n=3	1.80 (0.56 - 5.81) n=3	2.02 (0.79 - 5.16) n=4		
Pharynx Carcinoma	**1.70 (1.03 - 2.81)** n=2		**1.96 (1.01 - 3.80)** n=1		**1.96 (1.02 - 3.80)** n=1	**1.62 (1.01 - 2.59)** n=2
Salivary Gland Carcinoma	0.71 (0.23 - 2.20) n=1	1.77 (0.56 - 5.60) n=1	1.20 (0.34 - 4.20) n=1			
SCC	**1.80 (1.36 - 2.36)** n=5	**1.88 (1.21 - 2.93)** n=2	**1.72 (1.04 - 2.86)** n=6		**0.27 (0.09 - 0.80)** n=1	
**Liver**	1.41 (0.98 - 2.03) n=4	**1.51 (1.26 - 1.81)** n=2	**1.39 (1.09 - 4.10)** n=1			
Cancer	**1.62 (1.08 - 2.44)** n=3	**1.51 (1.26 - 1.81)** n=2	**1.39 (1.09 - 4.10)** n=1			
Cholangiocarcinoma	0.84 (0.51 - 1.40) n=1					
**Lung**	**1.57 (1.06 - 2.33)** n=8	**1.87 (1.27 - 2.74)** n=3		1.75 (0.59 - 5.15) n=3		
Adenocarcinoma	**1.83 (1.10 - 3.10)** n=1					
Mesothelioma				0.77 (0.31 - 1.90) n=1		
Non-Small Cell Lung Cancer	**1.84 (1.02 - 3.33)** n=4	**1.87 (1.27 - 2.74)** n=3		2.46 (0.71 - 8.56) n=2		
Small Cell Lung Cancer	1.04 (0.58 - 1.90) n=1					
SCC	1.28 (0.41 - 3.98) n=2					
**Ovary**	1.42 (0.82 - 2.45) n=4					1.24 (0.67 - 2.30) n=1
Cancer	1.16 (0.45 - 2.99) n=2					
Serous Carcinoma	1.82 (0.99 - 3.30) n=1					1.24 (0.67 - 2.30) n=1
Serous Cystadenocarcinoma	1.86 (0.85 - 4.00) n=1					
**Pancreas**	**2.37 (1.04 - 5.43)** n=5	2.98 (0.56 - 15.9) n=2		**1.49 (1.07 - 2.10)** n=1		
Adenocarcinoma	**1.60 (1.12 - 2.29)** n=3					

Organ and Tumor Type	OS	DFS	LC	DSS	MFS	PFS
Cancer	1.07 (0.63 - 1.80)<br>n=1					
Ductal Carcinoma		1.33 (0.97 - 1.80)<br>n=1		**1.49 (1.07 - 2.10)**<br>n=1		
Neuroendocrine Tumor	**35.3 (10.3 - 121)**<br>n=1	**7.36 (3.11 - 17.0)**<br>n=1				
**Penis**		1.35 (0.55 - 3.30)<br>n=1				
SCC		1.35 (0.55 - 3.30)<br>n=1				
**Small Intestine**	2.58 (0.21 - 31.8)<br>n=2					
Cancer	0.73 (0.51 - 1.10)<br>n=1					
Neuroendocrine Tumor	**9.47 (4.6 - 19.5)**<br>n=1					
**Soft tissue**	**2.97 (1.65 - 5.34)**<br>n=2	**3.41 (1.58 - 7.30)**<br>n=1		**1.65 (1.11 - 2.45)**<br>n=2	1.65 (0.72 - 3.80)<br>n=1	
Sarcoma	**2.97 (1.65 - 5.34)**<br>n=2	**3.41 (1.58 - 7.30)**<br>n=1		**1.65 (1.11 - 2.45)**<br>n=2	1.65 (0.72 - 3.80)<br>n=1	
**Stomach**	**1.92 (1.39 - 2.67)**<br>n=3	1.27 (0.77 - 2.10)<br>n=1				
Adenocarcinoma	**5.59 (1.22 - 26.0)**<br>n=1					
Cancer	1.83 (1.30 - 2.56)<br>n=2	1.27 (0.77 - 2.10)<br>n=1				
**Vulva**		1.52 (0.79 - 2.90)<br>n=1		1.34 (0.67 - 2.70)<br>n=1	**2.25 (1.42 - 3.60)**<br>n=1	
SCC		1.52 (0.79 - 2.90)<br>n=1		1.34 (0.67 - 2.70)<br>n=1	**2.25 (1.42 - 3.60)**<br>n=1	
