# Supplementary material for: Prognostic Significance of Carbonic Anhydrase IX Expression in Cancer Patients: A Meta-Analysis
Source: Front Oncol. 2016 Mar 29;6:69. doi: 10.3389/fonc.2016.00069 (PMC4810028; doi:10.3389/fonc.2016.00069)
Supplement: Supplementary file 3 [file Data_Sheet_1.pdf]

Supplementary File 1. Literature search strategy to identify papers for the meta-analysis that describe an association between tumoral CAIX expression and prognosis.

Research question: What is the prognostic value of tumoral CAIX expression in patients with solid tumors?

Keywords identified as search terms:
CAIX, Tumor, and Prognosis

Search algorithm Pubmed:
Prognosis:
(Prognos*) OR (surviv*) OR (hazard) OR (disease-free) OR (“disease free”) OR (progression-free) OR (“progression free”) OR (Kaplan-meier) OR (“Kaplan meier”) OR (predict*) OR (outcome) OR (efficacy) OR (effective*)
Hits: 4,589,956

CAIX:
(CAIX) OR (ca9) OR (“carbonic anhydrase IX”) OR (“carbonic anhydrase 9”) OR (“carbonic anhydrase-IX”) OR (“carbonic anhydrase-9”) OR (CA-IX) OR (ca-9) OR (G250)
Hits: 2,419

Tumor:
(tumor) OR (tumors*) OR (tumor’s) OR (tumoral*) OR (“tumor associated”) OR (tumor-associated) OR (“tumor related”) OR (tumor-related) OR (tumorigen*) OR (tumorous*) OR (tumour*) OR (cancer) OR (cancers) OR (cancer’s) OR (cancerogen*) OR (cancera*) OR (cancer-associate*) OR (cancerigen*) OR (cancerno*) OR (cancero*) OR (cancerp*) OR (“cancer related”) OR (cancer-related) OR (*carcinoma) OR (*sarcoma) OR (neoplas*) OR (malignanc*) OR (melanoma)
Hits: 3,618,127

Prognosis AND CAIX AND Tumor:
((Prognos*) OR (surviv*) OR (hazard) OR (disease-free) OR (“disease free”) OR (progression-free) OR (“progression free”) OR (Kaplan-meier) OR (“Kaplan meier”) OR (predict*) OR (outcome) OR (efficacy) OR (effective*)) AND ((CAIX) OR (ca9) OR (“carbonic anhydrase IX”) OR (“carbonic anhydrase 9”) OR (“carbonic anhydrase-IX”) OR (“carbonic anhydrase-9”) OR (CA-IX) OR (ca-9) OR (G250)) AND ((tumor) OR (tumors*) OR (tumor’s) OR (tumoral*) OR (“tumor associated”) OR (tumor-associated) OR (“tumor related”) OR (tumor-related) OR (tumorigen*) OR (tumorous*) OR (tumour*) OR (cancer) OR (cancers) OR (cancer’s) OR (cancerogen*) OR (cancera*) OR (cancer-associate*) OR (cancerigen*) OR (cancerno*) OR (cancero*) OR (cancerp*) OR (“cancer related”) OR (cancer-related) OR (*carcinoma) OR (*sarcoma) OR (neoplas*) OR (malignanc*) OR (melanoma))
Hits: 940

Search algorithm Embase:
Prognosis:
(Prognos$) OR (surviv$) OR (hazard) OR (disease-free) OR ("disease free") OR (progression-free) OR ("progression free") OR (Kaplan-meier) OR ("Kaplan meier") OR (predict$) OR (outcome) OR (efficacy) OR (effective$)
Hits: 5,100

CAIX:
(CAIX) OR (ca9) OR ("carbonic anhydrase IX") OR ("carbonic anhydrase 9") OR ("carbonic anhydrase-IX") OR ("carbonic anhydrase-9") OR (CA-IX) OR (ca-9) OR (G250)
Hits: 512

Tumor:
(tumor) OR (tumors$) OR (tumor's) OR (tumoral$) OR ("tumor associated") OR (tumor-associated) OR ("tumor related") OR (tumor-related) OR (tumorigen$) OR (tumorous$) OR (tumour$) OR (cancer) OR (cancers) OR (cancer's) OR (cancerogen$) OR (cancera$) OR (cancer-associate$) OR (cancerigen$) OR (cancerno$) OR (cancero$) OR (cancerp$) OR ("cancer related") OR (cancer-related) OR ($carcinoma) OR ($sarcoma) OR (neoplas$) OR (malignanc$) OR (melanoma)
Hits: 3291

Prognosis AND CAIX AND Tumor:
((Prognos$) OR (surviv$) OR (hazard) OR (disease-free) OR ("disease free") OR (progression-free) OR ("progression free") OR (Kaplan-meier) OR ("Kaplan meier") OR (predict$) OR (outcome) OR (efficacy) OR (effective$)) AND ((CAIX) OR (ca9) OR ("carbonic anhydrase IX") OR ("carbonic anhydrase 9") OR ("carbonic anhydrase-IX") OR ("carbonic anhydrase-9") OR (CA-IX) OR (ca-9) OR (G250)) AND ((tumor) OR (tumors$) OR (tumor's) OR (tumoral$) OR ("tumor associated") OR (tumor-associated) OR ("tumor related") OR (tumor-related) OR (tumorigen$) OR (tumorous$) OR (tumour$) OR (cancer) OR (cancers) OR (cancer's) OR (cancerogen$) OR (cancera$) OR (cancer-associate$) OR (cancerigen$) OR (cancerno$) OR (cancero$) OR (cancerp$) OR ("cancer related") OR (cancer-related) OR ($carcinoma) OR ($sarcoma) OR (neoplas$) OR (malignanc$) OR (melanoma))
Hits: 18

Literature search was performed in Pubmed and Embase on the 31th of August 2015.
