# Supplementary material for: Prognostic Significance of Carbonic Anhydrase IX Expression in Cancer Patients: A Meta-Analysis
Source: Front Oncol. 2016 Mar 29;6:69. doi: 10.3389/fonc.2016.00069 (PMC4810028; doi:10.3389/fonc.2016.00069)
Supplement: Supplementary file 4 [file Data_Sheet_2.pdf]

Supplementary File 2. Adjusted version of the Newcastle-Ottawa Scale to assess the quality of the included papers. In brief, for each criteria a single option can be registered. The amount of answers with stars behind them are counted and the total number of stars is a measurement of the study quality.

NEWCASTLE – OTTAWA QUALITY ASSESSMENT SCALE
Adjusted version

Note: A study can be awarded a maximum of one star for each numbered. When criteria are not reported no star can be awarded for that category.

First author: ....**Year of publication:** ………………

Selection

1) Representativeness of the cohort
    a. Truly representative of the average patient population ★
    b. Somewhat representative of the average patient population ★
    c. Selected group of patients based on certain criteria
    d. No description of the derivation of the cohort

Grouping variable

1) Was the measurement performed blindly from the outcome
    a. Yes ★
    b. No
    c. Not stated
2) How many persons performed the scoring
    a. More than one ★
    b. One
    c. Not stated
3) Was scoring between investigators performed independently
    a. Yes ★
    b. No
    c. Not stated

Outcome

1) Assessment of outcome
    a. Independent blind assessment ★
    b. Record linkage ★
    c. Self-report
    d. No description
2) Was follow-up long enough for outcomes to occur
    a. Yes ★
    b. No

3) <u>Adequacy of follow-up of cohorts</u>

   a. Complete follow-up – all subjects accounted for ★
   b. Subjects lost to follow-up unlikely to introduce bias ★
      (Equally distributed/small numbers)
   c. Follow-up rate unevenly distributed or large numbers lost
   d. No statement
