# Supplementary material for: Prognostic Significance of Carbonic Anhydrase IX Expression in Cancer Patients: A Meta-Analysis
Source: Front Oncol. 2016 Mar 29;6:69. doi: 10.3389/fonc.2016.00069 (PMC4810028; doi:10.3389/fonc.2016.00069)

Supplementary Figure S1. Funnel plots of papers reporting on OS (A), DFS (B), LC (C), DSS (D), MFS (E), and PFS (F). The x-axis represents the HR for each paper with the corresponding standard error on the y-axis. Heterogeneity between studies is present when papers are plotted outside the displayed funnel. Heterogeneity is significant for OS (A), DFS (B), LC (C), DSS (D), and MFS (E). For PFS (F) the lack of papers in the bottom right part of the funnel is suggestive of publication bias.

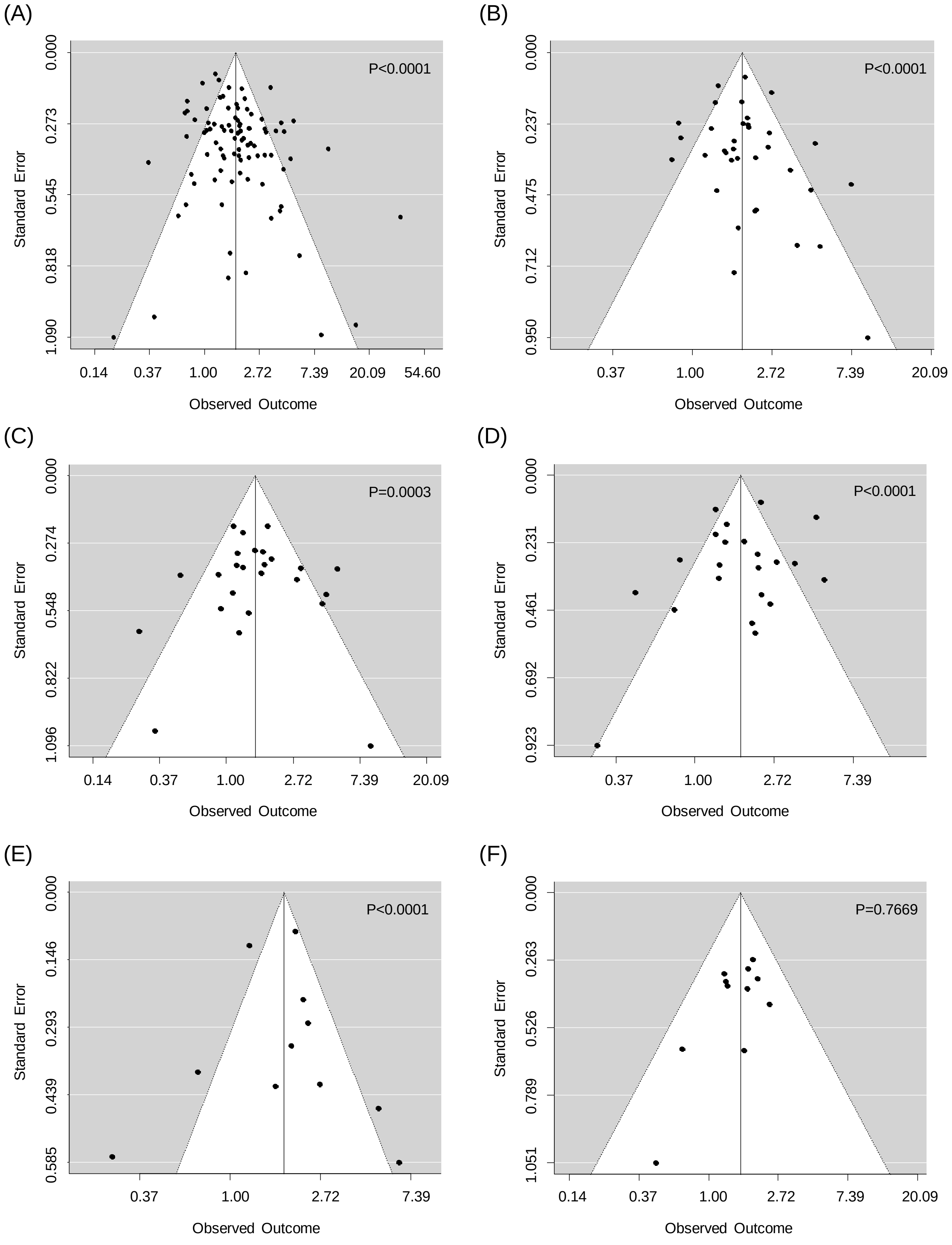

Supplement: Supplementary file 5 [file Image_1.pdf]
